# Medication-Related Osteonecrosis of the Jaws Initiated by Zoledronic Acid and Potential Pathophysiology

**DOI:** 10.3390/dj9080085

**Published:** 2021-07-30

**Authors:** Aya Alsalih, Annica Dam, Pia Lindberg, Anna Truedsson

**Affiliations:** 1 Faculty of Odontology, Malmö University, 205 06 Malmö, Sweden; aya.alsalih@gmail.com (A.A.); dam.annica@gmail.com (A.D.); 2 Department of Oral Pathology, Faculty of Odontology, Malmö University, 205 06 Malmö, Sweden; pia.lindberg@mau.se; 3 Department of Oral Maxillofacial Surgery and Oral Medicine, Faculty of Odontology, Malmö University, 205 06 Malmö, Sweden

**Keywords:** medication-related osteonecrosis of the jaw, MRONJ, pathophysiology, zoledronate, zoledronic acid

## Abstract

The aim of this systematic review is to present an up-to-date review of available publications investigating the cellular mechanisms initiating the development of medication-related osteonecrosis of the jaw caused by zoledronic acid. Electronic searches of MEDLINE/PubMed and Scopus were conducted on the 3 June 2019. A total of 804 publications were identified, of which 11 met the inclusion criteria and were, therefore, included in this study. All the included studies were in vitro studies investigating various human cells. The current review found that zoledronic acid in various concentrations increased apoptosis and decreased migration and proliferation of epithelial cells, fibroblasts, osteoblasts, endothelial cells and dental pulp stem cells, which can affect local tissue homeostasis. The consequences of zoledronic acid were found to be both time- and dose-dependent. The pathophysiology of medication-related osteonecrosis of the jaw is likely a multifactorial process involving prolonged wound healing, chronic inflammation and altered bone remodelling following the administration of zoledronic acid. Further research is needed to identify the exact pathophysiology to optimise management and treatment.

## 1. Introduction

Reports of osteonecrosis of the jaw in patients receiving bisphosphonate (BP) therapy were first described by Marx in 2003 [1]. In recent years, osteonecrosis of the jaw has been observed to be a side effect of various medications used to treat osteoporosis, preventing skeletal-related events, various cancers and bone metastases. Therefore, the medical term used today is ‘medication-related osteonecrosis of the jaw’ (MRONJ). The medications included in the American Association of Oral and Maxillofacial Surgeons (AAOMS) position paper include antiresorptive preparations (BPs), antiangiogenic medications and targets of the vascular endothelial growth factor pathway (tyrosine kinase inhibitor, humanised monoclonal antibody and mammalian target of rapamycin pathway) [2]. Other medications, such as radiopharmaceuticals, selective estrogen receptor modulators and immunosuppressants, have been documented to cause MRONJ [3]. Tocilizumab, used in the treatment of COVID-19, has also been suggested to cause MRONJ [4].

The most recent definition of MRONJ was set by the American Association of Oral and Maxillofacial Surgeons in *Medication-Related Osteonecrosis of the Jaw—2014 Update.* According to the AAOMS, the following can be indications of MRONJ: current or previous treatment with antiresorptive or antiangiogenic agents; exposed bone or bone that can be probed through an intraoral or extraoral fistula(e) in the maxillofacial region that has persisted for more than eight weeks; and no history of radiation therapy to the jaws or obvious metastatic disease to the jaws [2].

However, the European task force on medication-related osteonecrosis of the jaw have in a recent workshop suggested a revision of AAOMS position paper on MRONJ. The task force group concluded that many patients with MRONJ go undiagnosed due to not meeting the AAOMS’s definition and classification criteria [5].

The prevalence of MRONJ was reported to be 0.043–0.1% amongst patients treated with oral BPs for osteoporosis and 1.03–3% amongst patients treated with intravenous BPs for metastatic diseases [6,7]. An estimation of treatment duration before the development of MRONJ is 70.5 months for oral BPs, 30 months for zoledronic acid, 18 months for pamidronic acid, and 15.8 months for subcutaneous denosumab. The MRONJ lesion ratio in the mandible to the maxilla was 3:1, and lesions were more common in the posterior regions [6]. A higher prevalence is observed in females, which can be due to primary diseases such as osteoporosis [2].

Many risk factors have been reported for initiating MRONJ, both dental and medical. The most commonly reported dental risk factors are tooth extractions, implant installations and periodontal disease, while the most common medical risk factors are chemotherapy, corticosteroids and tobacco smoking. It is not yet clear why these are risk factors, but it has been speculated to be due to the involvement of local inflammation/infection and immunosuppression. However, diabetes, cardiovascular disease, local trauma and potentially poor oral hygiene have also been suggested as risk factors. It has also been reported that the risk for MRONJ lesion development increases with higher doses and prolonged treatment [8,9]. The European task force on MRONJ have debated whether tooth extraction per se is a risk factor for MRONJ, suggesting that the underlying infection leading to tooth extraction is of greater significance as a risk factor [4].

MRONJ pathophysiology is not yet identified, making it difficult to establish prophylactic and management guidelines. Different theories have been proposed. The AAOMS have mentioned inhibition of osteoclastic bone resorption and remodelling, inhibition of angiogenesis, inflammation/infection and soft tissue toxicity as possible mechanisms. The effects of bisphosphonates, impaired osteoclast function and survival as well as apoptosis of osteoclasts result in decreased resorption and remodelling of the bone, thus affecting bone healing [2]. Impaired bone remodelling and healing along with the toxic effects of bisphosphonates may play a role in the pathophysiology of MRONJ [10]. Another theory involves inhibition of angiogenesis supported by in vitro studies demonstrating reduced angiogenesis after zoledronic acid exposure and decreased levels of vascular endothelial growth factor (VEGF) in cancer patients treated with zoledronic acid. This implies that decreased angiogenesis leads to impaired vascular supply of the bone and, thereby, necrosis [2,10]. As mentioned, the most commonly reported dental risk factors are tooth extractions and periodontal disease [9], which may indicate involvement of inflammation and/or bacterial infection in the pathophysiology of MRONJ. Rats treated with BPs such as zoledronic acid (ZA) and alendronate developed MRONJ lesions in areas affected by periodontal disease and/or periapical lesions. This suggests that the development of MRONJ is accompanied by inflammation and/or infection. Although osteoclasts are the main target of bisphosphonates, these medications have been reported to affect the soft tissue cells. In vitro studies exposing oral epithelial cells to bisphosphonates reported decreased proliferation and increased apoptosis demonstrating toxic effects. These findings suggest involvement of soft tissue toxicity in the pathophysiology of MRONJ [2,10].

ZA is a commonly used nitrogen-containing bisphosphonate (N-BPs) with the highest affinity to hydroxyapatite crystals, primarily given in high doses for treatment of bone metastases [2]. The antiresorptive effects of N-BPs are partly due to their ability to inhibit the dissolution of hydroxyapatite crystals [11] and partly through their effects on the bone remodelling process. Bone remodelling is initiated by the recruitment and activation of osteoclast precursors by RANKL produced by osteoblasts. The activated osteoclasts form a resorption cavity and begin secreting H^+^ and Cl^−^ as well as enzymes leading to bone resorption [12]. Newly recruited osteoblasts begin synthesising and secreting osteoid, which is then mineralised into mature bone [13]. N-BPs disturb the bone remodelling process by inhibiting the peroxisomal enzyme—farnesyl pyrophosphate synthase—involved in the mevalonate pathway, resulting in impaired osteoclast function and survival, eventually leading to cell apoptosis and impaired bone resorption [14].

A review including 599 studies reported an increasing association between ZA treatment and MRONJ development [15]. It has also been reported that treatment timespan leading to MRONJ development was significantly shorter for ZA compared to other BPs, presumably due to its high affinity to hydroxyapatite crystals, which in turn leads to higher potency compared to other BPs [16]. Due to the increasing and aging population, it is predicted that the use of BPs and eventually MRONJ prevalence will also increase. Considering that this condition has devastating consequences for those affected, the need to understand the underlying mechanisms leading to MRONJ lesion development initiated by ZA is urgent. The aim of this study is to review available publications concerning cellular pathophysiology and identify the need for future research. Furthermore, MRONJ pathophysiology aids the practicing clinician in the risk assessment and treatment of existing lesions [10].

## 2. Materials and Methods

### 2.1. Protocol and Eligibility Criteria

This systematic review was carried out in accordance with the criteria of the Preferred Reporting Items for Systematic Reviews and Meta-Analyses (PRISMA) guidelines [17], using a predefined search and review protocol (see Supplementary Materials Table S1). The review question of the study was formed using the PICO Model:

(P) Population: Human cells.

(I) Intervention: Zoledronic acid’s effects on cellular mechanisms in bone remodelling and wound healing.

(C) Control: None.

(O) Outcome: Medication-related osteonecrosis of the jaw diagnosed according to the American Association of Oral and Maxillofacial Surgery criteria (previously known as bisphosphonate-related osteonecrosis of the jaw) [2].

The inclusion criteria for the included studies were as follows:

(1) In vitro studies.
(2) Publications in the English language.
(3) Only human cells.
(4) MRONJ pathophysiology must be the aim of the study.
(5) Associations to MRONJ must be made in the discussion.

The exclusion criteria for study selection were as follows:

(1) Osteonecrosis of the jaw not related to medication.
(2) Osteonecrosis in other body parts.
(3) Studies including bisphosphonates other than zoledronic acid.
(4) Zoledronic acid in combination with other medications.
(5) Systematic reviews, etiological studies, case series and reports, consensus reports, letters, editorials, doctoral theses, pilot studies and only abstracts.
(6) Studies investigating possible risk factors (i.e., different diseases).
(7) Comparative studies between different kinds of bisphosphonates.

### 2.2. Information Sources and Search

A systematic search was carried out in the electronic databases MEDLINE/PubMed and Scopus. The systematic search consisted of three blocks (the jaws, zoledronic acid and MRONJ). All three search blocks included both free-text words/phrases and Medical Subject Headings (MeSH) terms.

The following search phrase was used in the MEDLINE/PubMed search applying the filter ‘English publications only’ on 3 June 2019:

((((((((((maxilla[Title/Abstract]) OR maxillary[Title/Abstract]) OR mandible[Title/Abstract]) OR mandibular[Title/Abstract]) OR jaw[Title/Abstract]) OR “Jaw”[Mesh]) OR “Mandible”[Mesh]) OR “Maxilla”[Mesh])) AND ((((((((((((“Bisphosphonate-Associated Osteonecrosis of the Jaw”[Mesh]) OR “Osteonecrosis”[Mesh]) OR ONJ[Title/Abstract]) OR medication-related osteonecrosis of the Jaw[Title/Abstract]) OR osteonecrosis[Title/Abstract]) OR osteonecrosis of the jaw[Title/Abstract]) OR MRONJ[Title/Abstract]) OR BRONJ[Title/Abstract]) OR Bisphosphonate-related Osteonecrosis of the Jaw[Title/Abstract]) OR Bisphosphonate-Associated Osteonecrosis of the Jaw[Title/Abstract]) OR BONJ[Title/Abstract]) OR BAONJ[Title/Abstract])) AND (((Zoledronic Acid[Title/Abstract]) OR Zoledronate [Title/Abstract]) OR “Zoledronic Acid”[Mesh]).

The following search phrase was used in the Scopus search applying the filter ‘English publications only’ on the 3 June 2019:

(TITLE-ABS (“medication-related Osteonecrosis of the Jaw”)) or (TITLE-ABS (“ONJ”)) or (TITLE-ABS (“osteonecrosis”)) or (TITLE-ABS (“osteonecrosis of the jaw”)) or (TITLE-ABS (“MRONJ”)) or (TITLE-ABS (“BRONJ”)) or (TITLE-ABS (“Bisphosphonate-related Osteonecrosis of the Jaw”)) or (TITLE-ABS (“Bisphosphonate-Associated Osteonecrosis of the Jaw”)) or (TITLE-ABS (“BONJ”)) or (TITLE-ABS (“BAONJ”)) and ((TITLE-ABS (“jaw”)) or (TITLE-ABS (“maxilla”)) or (TITLE-ABS (“maxillary”)) or (TITLE-ABS (“mandible”)) or (TITLE-ABS (“mandibular”)) and (TITLE-ABS (“zoledronate”)) or (TITLE-ABS (“zoledronic acid”)) and (LIMIT-TO (LANGUAGE, “English”)).

### 2.3. Study Selection

**Figure 1:**
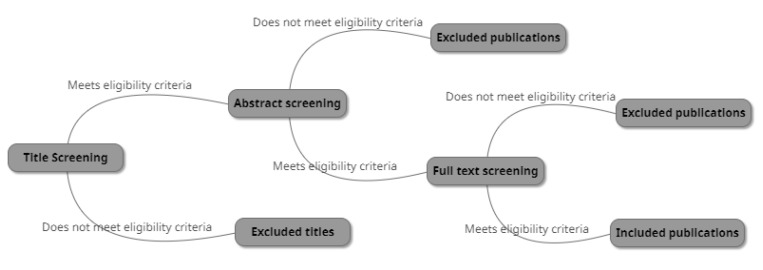
The selection process.

Identified publications from the conducted searches in MEDLINE/PubMed and Scopus were transferred to the Systematic Reviews web application, Rayyan QCRI [18] and reviewed by two investigators—A.A and A.D—independently. The titles were screened according to the eligibility criteria, and titles meeting the set criteria were further investigated by screening the abstracts. Abstracts deemed relevant to the eligibility criteria were further investigated in the full text. Publications that did not meet the eligibility criteria after a full-text screening were excluded. Inconsistencies were resolved by discussion and consensus between the two investigators (see Figure 1).

### 2.4. Data Extraction

The two investigators—A.A and A.D—independently collected data from the selected publications. The following data were collected and documented in tables: authors and year of publication, cell type, methods used and relevant reported findings.

### 2.5. Risk of Bias Assessment

The two authors—A.A and A.D—evaluated the quality of the included articles independently. The risk of bias assessment was determined using a variation based on the ToxR tool [19]. The chosen articles were assessed according to 15 quality parameters (identified test substance, test substance concentration, cell type and line, cells of oral origin, source of the cells used, controls of the same cell line, number of passages of cell lines, type/composition of medium used, method of administration, duration of exposure and time-point of observation, detection kits (origin and manufacturer), number of replicates, statistical methods for data analysis, clear methods and endpoints and transparent endpoint description). Each parameter fulfilled/mentioned gave a point. A total of 15–13 fulfilled parameters were classified as ‘reliable without restrictions’, 12–9 fulfilled parameters were classified as ‘reliable with restrictions’ and 8–1 fulfilled parameters were classified as ‘not reliable’.

### 2.6. Strategy for Data Synthesis

The two investigators—A.A and A.D—independently collected data from the selected publications. The data collected from the included publications were compiled and summarised. The potential cellular mechanisms of the pathophysiology of MRONJ induced by zoledronic acid were investigated and documented based on the present knowledge and published research.

## 3. Results

From both searches, 535 were duplicates. A total of 15 full-text articles were excluded from the study selection, see Supplementary Materials Table S2 for the reasons for exclusion.

### 3.1. Study Selection

**Figure 2:**
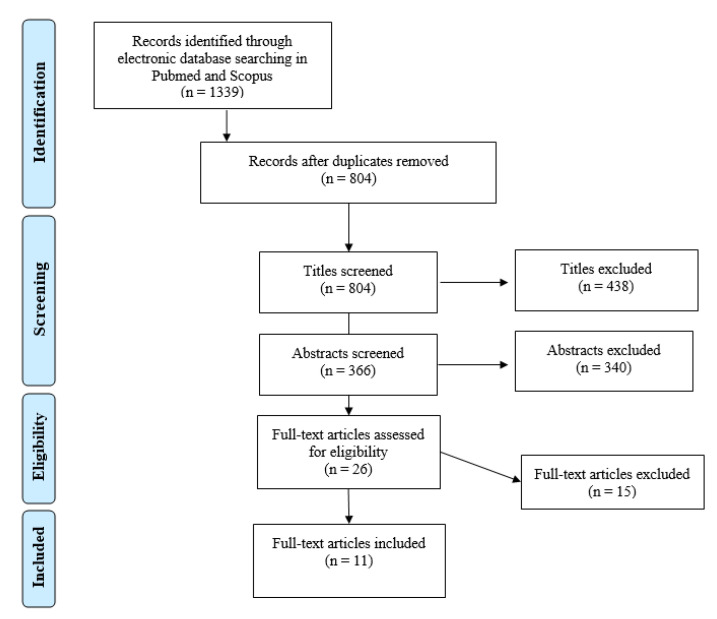
PRISMA flow diagram, in which some titles and abstracts were excluded due to not meeting the eligibility criteria (e.g., animal studies, systematic reviews, several BPs, case reports, etc.).

A total of 11 publications were included in this review (see Figure 2). All of the included studies were in vitro studies: five studies concern oral epithelial cells [20,21,22,23,24], six studies are about oral fibroblasts [20,21,22,25,26,27], three studies are about endothelial cells [23,28,29], three studies are about osteoblasts [24,26,30] and one study is about dental pulp stem cells (DPSC) [25]. The included 11 articles were further quality-assessed according to the aforementioned model. Two articles were deemed ‘reliable without restrictions’, while the remaining nine articles were ‘reliable with restrictions’ (see Table 1).

### 3.2. ZA Application Methods

Scheper et al. used dentine discs (DDs) as a means of applying ZA to oral epithelial cells mimicking ZA release from bone to oral cells. The DDs were either left unchelated or chelated with EDTA or EGTA to stimulate the effect saliva has on ZA release from bone in the oral environment [20]. Saracino et al. had osteoblasts cultured in medium conditioned (CM) by epithelial cells treated with various concentrations of ZA to inspect whether factors released by ZA-treated epithelial cells can affect osteoblast activity and, therefore, initiate MRONJ [24]. Komatsu et al. used various concentrations of ZA in infusion solution applied in combination with TGF-β to study ZA’s effect on TGF-β-induced fibroblast differentiation during inflammation [27]. The remaining eight articles used cultured medium or infusion solution supplemented with various concentrations of ZA as the method of direct application to cells.

The included material and their reported results are presented in Table 2 and Table 3.

## 4. Discussion

Different theories about the pathophysiology of MRONJ have been proposed in the position paper *Medication-Related Osteonecrosis of the Jaw—2014 Update (AAOMS).* Inhibition of osteoclastic bone resorption and remodelling, inhibition of angiogenesis, inflammation/infection and soft tissue toxicity are mentioned as possible mechanisms [2]. The purpose of this study was to review available publications investigating the possible cellular processes leading to MRONJ development.

Three publications investigated the role of osteoblasts in bone remodelling and their contribution to MRONJ development. High ZA concentrations were shown to both decrease proliferation levels [24,26,30] and increase apoptosis [26] in osteoblasts. Furthermore, medium conditioned by ZA-treated epithelial cells was able to decrease proliferation and expression of factors (BMP-4, TGF-β2 and ALP) in osteoblasts, suggesting soft tissue involvement in activation of bone remodelling and reduced bone formation ability (A7). RANKL expression was also shown to be increased by the conditioned medium proposing impaired bone remodelling, which in turn may eventually lead to or play a role in MRONJ development [24].

None of the included publications investigated the effect of ZA on osteoclasts. This may be because osteoclasts are the target cells for BPs’ pharmacological effects, and therefore, the osteoclastic involvement in MRONJ pathophysiology is not of great interest. However, considering that osteoclasts play a major role in bone remodelling, their involvement in MRONJ pathophysiology should be investigated.

Three of the included publications investigated the involvement of endothelial cells and ZA’s antiangiogenic effect in the pathophysiology of MRONJ [23,28,29]. Collectively, ZA was shown to increase apoptosis and decrease viability, which is in agreement with previous studies [31]. The ZA-induced apoptosis of endothelial cells could be due to increased autophagic activity [29]. A6 and A8 have also reported that ZA suppressed endothelial cell migration and angiogenesis [23]. A8 reported the accumulation of endothelial cells in the S phase suggesting an altered cell cycle and thereof suppressed cell proliferation [28]. Angiogenesis is an important process in tissue repair and inflammation, and both cell migration and proliferation are heavily involved in this process [32,33]. The reported ZA-related angiogenic effects propose its engagement in impaired or possibly delayed wound healing and bone remodelling that can eventually lead to and maintain MRONJ, which is in agreement with previous studies [2,10,31,34].

The majority of the included publications in this review investigated the effect of ZA on oral epithelial cells and fibroblasts. An increase in apoptosis and a decrease in proliferation, viability and migration was reported for both oral epithelial cells and fibroblasts. A7 reported an increase in pro-inflammatory factors (TNF-α) and a decrease in anti-inflammatory factors (PPAR-α), suggesting that ZA triggers the pro-inflammatory potential in oral epithelial cells [24]. A2 and A11 reported a ZA-induced inhibition of collagen type I deposition by oral fibroblasts [27]. A9 reported an increase in ratio of phosphorylated NF-κB/NF-κB after treatment with ZA, suggesting an increased expression of several pro-inflammatory factors and an eventual chronic inflammation [26,35]. Considering that the oral cavity has the unique feature of the oral epithelial lining being close to the underlying bone [36], continuously released BPs from the bone can easily affect oral tissue cells [37]. A recent review by Chang et al. have also reported similar findings regarding the effect of ZA on both fibroblasts and endothelial cells [38].

A11 reported that ZA treatment reversed the effects of TGF-β on oral fibroblasts as well as suppressing collagen type I expression [27]. TGF-β is involved in the wound healing process by promoting fibroblast differentiation into myofibroblasts, migration and increased viability [39]. Cell migration, proliferation and collagen deposition are essential to the wound healing process, and disruption in these processes lead to defective repair, which in turn can be a contributing factor to MRONJ.

Only one of the 11 included publications investigated the effect of ZA treatment on DPSCs. Apoptosis was reported to be increased, while proliferation and viability were decreased [25]. DPSCs are a type of mesenchymal stem cell in the oral cavity and can represent other oral stem cells. The differentiation of stem cells into different types of cells is of importance in the wound healing process [40]. However, it is important to take into account that only one study discussed the involvement of these cells, and therefore, no conclusions could be made.

It is known that the hard tissue plays a role as a ZA reservoir, from which it is theorised to be continuously released [41,42]. The negative effects of ZA on soft tissue cells, such as oral epithelial cells and fibroblasts, can cause thinning of the oral mucosa covering the hard tissue exposing bone to the oral environment. Dental trauma (tooth extraction, etc.) can also lead to exposed bone in the oral cavity. The exposed tissue is now more vulnerable to infections. This, in combination with the aforementioned impaired angiogenesis and chronic inflammation, leads to prolonged wound healing and possibly a secondary infection and eventual necrosis of the bone. This is partially supported by the reported findings in the included studies.

Two of the included publications used unconventional ZA-application methods, while the remaining nine publications used cultured medium or infusion solution supplemented with various concentrations of ZA as the method of direct application to cells. The difference in application method might be an explanation as to why greatly varying ZA-concentrations gave the same effect. As mentioned, A1 used dentine disks that were either chelated or non-chelated [20]. The chelated model induced apoptosis in both oral fibroblasts and epithelial cells at lower ZA-concentrations, this advocates for the hypothesis that the chelating activity of saliva on bone releases ZA, and therefore, causes soft tissue damage due to higher concentrations locally.

In A7, osteoblasts were cultured in medium conditioned by epithelial cells treated with various concentrations of ZA. The osteoblasts showed decreased proliferation; however, direct application of ZA also led to the same results at similar concentrations (see Supplementary Materials Table S3), suggesting that the decreased proliferation is a result of the ZA itself and not significant to medium conditioned by ZA-treated epithelial cells. On the other hand, the under-expression of factors involved in bone remodelling was a unique finding for the CM, suggesting soft tissue involvement in the activation of bone remodelling and reduced bone formation ability, as mentioned earlier [24].

In vitro studies usually focus on a single type of cell in a laboratory environment, while the reality is a complex combination of tissues in near proximities interacting and influencing each other. It should not be disregarded that the oral cavity also has the significant feature of hosting diverse microflora and saliva. This emphasises the importance of developing an in vitro model including the different types of cells involved and their interactions. In vivo studies reflect the reality but can compromise patient safety and possibly violate ethical guidelines.

A vast spectrum of different ZA concentrations has been used in the included publications, ranging from lowest to highest from 0.1 up to 500 μM. As evident by the reported results, oral epithelial cells, DPSCs and fibroblasts were more susceptible to the effects of ZA compared to osteoblasts. No explanation for these results was found in this study; however, these findings support the aforementioned theory suggesting bone exposure due to initial thinning of soft tissue and dental trauma, resulting in secondary bone necrosis.

According to pharmacokinetic studies, the ZA concentration after infusion (4 mg administered for 15 min) is calculated to be approximately 1µM/L. Bone concentrations, on the other hand, are thought to be >100-fold higher and sustained for at least six months after infusion [42]. Considering mastication and teeth bearing, the jaws have a higher bone turnover rate and richer blood supply compared to other osseous parts, resulting in bisphosphonate accumulation, which can explain the dominant prevalence of osteonecrosis in the jaws [41,43]. The bone-bound ZA is believed to be slowly released over time to the surrounding tissues [42]. The amount of released ZA is currently not known; therefore, it is difficult to predict which concentrations of ZA are relevant for the pathophysiology of MRONJ, which can explain the vast spectrum of different concentrations used. This indicates the necessity for future research into the actual amounts of ZA released in the surrounding tissues in relation to the development of MRONJ.

A universal finding in the included publication is that the effects of ZA on the different cell types were both dose- and time-dependent (see Supplementary Materials Tables S4–S8). This may explain the increasing prevalence of MRONJ with increased dosage and treatment span, advocating that these two parameters are risk factors for the development of MRONJ.

## 5. Conclusions

The present study explored the effects of zoledronic acid on various cells of the oral cavity and the possible underlying cellular mechanisms involved in the pathophysiology of medication-related osteonecrosis of the jaws. The current findings show negative effects following ZA administrations, including increased cell apoptosis, and decreased migration and proliferation, leading to prolonged wound healing, chronic inflammation and altered bone remodelling. These effects have also been shown to be both dose- and time-dependent. This suggests a multifactorial pathophysiology influenced by different cellular processes, anatomical relations, patients’ medical and dental histories and risk for infection. Considering the growing population and prolonged lifespan, the incidence of MRONJ is expected to increase, thus highlighting the need for future research to elicit the definitive pathophysiology and the development of appropriate risk-assessment and treatment measures, as this condition has devastating consequences for those affected.

## Figures and Tables

**Table 1:** Risk of bias assessment results. (**A**) Identified test substance, (**B**) test substance concentration, (**C**) cell type and line, (**D**) cells of oral origin, (**E**) source of the cells used, (**F**) controls of the same cell line, (**G**) number of passages of cell lines, (**H**) type/composition of medium, (**I**) method of administration, (**J**) duration of exposure and time-point of observation, (**K**) detection kits (origin and manufacturer), (**L**) number of replicates, (**M**) statistical methods for data analysis, (**N**) clear methods and endpoints and (**O**) transparent endpoint description.

Article (A)	A	B	C	D	E	F	G	H	I	J	K	L	M	N	O	Overall Score
A1 Scheper et al., 2010 [20].	1	1	1	0	0	1	0	1	1	1	1	1	1	1	1	12
A2 Ravosa et al., 2016 [21].	1	1	1	1	0	1	0	1	1	1	1	1	1	1	1	13
A3 Pourgonabadi et al., 2017 [25].	1	1	1	1	0	1	1	1	0	1	1	0	1	0	1	11
A4 Scheper et al., 2009 [22].	1	1	1	0	0	1	0	1	1	1	1	1	1	1	1	12
A5 Thibaut et al., 2014 [30].	1	1	1	0	1	1	0	1	1	1	1	1	1	1	0	12
A6 Wang et al., 2019 [23].	1	1	1	0	1	1	0	1	0	1	1	0	1	1	1	11
A7 Saracino et al., 2012 [24].	0	1	1	0	1	1	0	1	1	1	1	0	1	1	0	10
A8 Lang et al., 2016 [28].	0	1	1	0	1	1	0	1	1	1	1	1	1	1	0	11
A9 Anitua et al., 2016 [26].	1	1	1	1	0	1	1	1	1	1	1	1	1	1	1	14
A10 Lu et al., 2016 [29].	1	1	1	0	0	1	0	1	1	1	1	1	1	1	1	12
A11 Komatsu et al., 2016 [27].	1	1	0	1	0	0	0	1	1	1	1	1	1	1	0	10

**Table 2:** Presentation of the included material and their reported results. For details about the methods used to acquire these results, see Supplementary Materials Table S3.

Article (A)	ZA-Concentration (µM)	Cell Type	Results
A1	0.5, 1, 3, 5, 10	Human gingival fibroblasts	- Dose-dependent increase in apoptosis in the chelated and non-chelated DDs starting at 0.5 µM and 1 µM respectively.
		Human Keratinocytes	- Dose-dependent increase in apoptosis in the chelated and non-chelated DDs starting at 0.5 µM and 3 µM, respectively. - Significant difference in the apoptosis percentage between the chelated and non-chelated DDs. - Non-significant difference in proliferation in the non-chelated DDs. However, a dose-dependent decrease in proliferation in the chelated DDs starting from 1 µM. - Significant decrease in proliferation starting at 0.5 µM in cells treated directly with ZA in infusion solution.
A2	5, 10, 30, 50, 75, 100, 300	Human oral Fibroblasts	- Dose-dependent significant increase in apoptosis starting from 10 µM. - Dose-dependent significant decrease in proliferation starting from 10 µM. - Significantly decreased rate of cell migration when treated with 10 µM starting from 40 h. - Cells treated with ZA at a concentration of 10 µM showed, at 48 h, that collagen expression was significantly inhibited, with a decrease ranging between 60–70% for collagen type I. Concurrently, collagen deposition was not apparent.
		Human oral epithelial cells	- Dose-dependent significant increase in apoptosis starting from 30 µM. - Dose-dependent significant decrease in proliferation starting from 10 µM. - Significantly increased rate of cell migration when treated with 10 µM starting from 10 h.
A3	0.2, 0.4, 0.8 1.5, 3, 6, 12, 25, 50, 100	Dental pulp stem cells (DPSCs)	- Dose-dependent significant increase in apoptosis starting from 0.8 µM at 72 h. - Dose-dependent significant decrease in proliferation starting from 0.8 μM at 72 h and 100 μM at 48 h. - Significant decrease in viability starting from 1.5 µM when treated for 72 h.
		Human gingival fibroblasts	- Dose-dependent significant decrease in proliferation starting from 0.8 μM when treated for 7 d and 1.5 μM at 72 h.
A4	0.25, 0.5, 1, 3	Human gingival fibroblasts	- Significant dose-dependent increase in apoptosis starting from 0.25 μM. - Dose-dependent significant decrease in proliferation starting from 1 µM.
		Human keratinocytes	- Dose-dependent significant increase in apoptosis starting from 0.25 μM. - Dose-dependent significant decrease in proliferation starting from 1 µM.
A5	0.1, 10	Human foetal osteoblasts (hFOB)	- Significant decrease in proliferation starting from 3 d when treated with 10 µM. - Significant increase in proliferation when treated with 0.1 µm for 10 days.
A6	5, 50, 100	Human umbilical vein endothelial cells	- Significant increase in apoptosis at concentrations starting from 50 µM. - Dose-dependent significant decrease in viability starting from 5 µM in a manner. - Significant inhibition of migration and angiogenesis at 5 µM (48 h).
		Human oral keratinocytes	- Significant increase in apoptosis at concentrations starting from 50 µM. - Dose-dependent significant decrease in viability starting from 5 µM. - Significant decrease in migration at 5 µM.
A7	5, 50 µM 5, 50 CM	Human keratinocytes	- Significant increase in apoptosis starting from 5 µM. - Significant decrease in proliferation starting from 5 µM. - Significant increase in TNF-⍺ levels and a decrease in PPAR⍺ expression levels starting from 5 µM when treated for 48 h.
		Human osteoblast-like cells	- Significant decrease in proliferation starting from 5 CM and when treated with 5 µM directly. - No significant effects on viability in any of the used concentrations. - Significant decrease in BMP-4 expression for 5 and 50 CM and ALP expression for 50 CM was observed. RANKL displayed a significant increase for 5 and 50 CM.
A8	0.23, 0.69 2.06, 6.17, 15, 18.52, 50, 55.56, 150, 166.67, 500	Human umbilical vein endothelial cells	- Significant increase in apoptosis starting from 15 µM. - Significant decrease in viability starting from 2.06 μM. - Dose-dependent, significant impairment of the cell cycle was evident as cells accumulated in the S phase and dissipated in G2/M phases at concentrations starting from 15 µM for 12 h. - Significant decrease in migration starting from 15 µM.
A9	0.1, 1, 2, 3, 4, 5, 10	Human gingival fibroblasts	- Significant increase in apoptosis starting from 10 µM. - The ratio between pNF-κB/NF-κB was significantly increased when incubated with 10 µM for 48 h.
	0.1, 1, 5, 10, 12.5, 15, 17.5, 50	Human alveolar osteoblasts	- Significant increase in apoptosis starting from 20 µM. - The ratio between pNF-κB/NF-κB was significantly increased when incubated with 25 µM for 6 h. - Dose-dependent significant decrease in proliferation starting from 12.5 μM (24 h) and starting from 10 μM (48–96 h).
A10	25, 50, 75, 100	Human umbilical vein endothelial cells	- Significant increase in apoptosis starting from 25 µM. - ZA-induced autophagy by increasing the autophagic activity. - Autophagy inhibition under ZA treatment-decreased apoptosis (100 µM). - Significant decrease in viability starting from 50 μM.
A11	0.147, 1.47, 14.7, 147	Human gingival fibroblasts	- Significantly suppressed viability starting at 14.7 µM. - ZA at 1.47 μM (48 h) suppressed the TGF-β type I-induced migratory activity. - ZA at 1.47 μM (48 h) suppressed the TGF-β type I-induced increase in cell viability. - ZA (1.47 μM) suppressed the upregulation of collagen type I expression levels induced by TGF-β type I.

**Table 3:** Cell processes investigated in the included articles (a summarised version of Table 2). + = increase, - = decrease, ns = not significant, x = not investigated. * Increased migration was investigated only at the lowest concentration. For more details, see Supplementary Materials Tables S4–S8.

Cell Type	Article (A)	Apoptosis	Proliferation	Viability	Migration
Epithelial cells	A1	+	-	x	x
	A2	+	-	-	(+) *
	A4	+	-	-	-
	A6	+	x	-	-
	A7	+	-	x	x
Fibroblasts	A1	+	x	x	x
	A2	+	-	-	-
	A3	x	-	x	x
	A4	+	-	x	x
	A9	+	x	-	x
	A11	x	x	-	x
Osteoblasts	A5	x	-	+/-	x
	A7	x	-	ns	x
	A9	+	x	x	x
Endothelial cells	A6	+	x	-	-
	A8	+	x	-	-
	A10	+	x	-	x
Dental pulp stem cells	A3	+	-	-	x

## Data Availability

Not applicable.

## Supplementary Materials

The following are available online at https://www.mdpi.com/article/10.3390/dj9080085/s1, Table S1. Review Protocol. Table S2. Excluded articles. Table S3. Methods used in the included publications. Table S4. Detailed presentation of results regarding epithelial cells. Table S5. Detailed presentation of results regarding fibroblasts. Table S6. Detailed presentation of results regarding osteoblasts. Table S7. Detailed presentation of results regarding endothelial cells. Table S8. Detailed presentation of results regarding dental pulp stem cells.
