# Supplementary material for: Medication-Related Osteonecrosis of the Jaws Initiated by Zoledronic Acid and Potential Pathophysiology
Source: Dent J (Basel). 2021 Jul 30;9(8):85. doi: 10.3390/dj9080085 (PMC8392270; doi:10.3390/dj9080085)
Supplement: Supplementary file 1 [file dentistry-09-00085-s001.zip › dentistry-1282530-supplementary.pdf]

Supplementary Materials

Table S1. Review Protocol.

The review protocol

Methods of the analysis and inclusion/exclusion criteria were specified in advance and documented in a protocol.

Review question

What are the possible cellular mechanisms involved in the pathophysiology of medication-related osteonecrosis of the jaw (MRONJ) initiated by zoledronic acid?

Searches

The first systematic search will be carried out in the database Pubmed using three search blocks.

- The first block will consist of the search phrases: medication-related osteonecrosis of the jaw OR MRONJ OR bisphosphonates-related osteonecrosis of the jaw OR BRONJ OR osteonecrosis of the jaw OR ONJ.
- The second block will consist of the active substance zoledronate or zoledronic acid which is mentioned on the AAOMS's list of medications related to osteonecrosis of the jaw.
- The third search block will consist of the search words: jaw OR maxilla OR mandible.

An advanced search will also be conducted using medical subject headings search (MeSH) terms for the search phrases that have established MeSH terms. The second systematic search will be carried out in the database Scopus using the search phrases listed above.

The database searches will be conducted and reviewed by both operators independently at the same time point. The identified publications from all the conducted searches will be combined and duplicate publications will be excluded.

Types of studies to be included

Studies to be included: (1) in vitro studies, (2) publications in the English language, (3) only human cells, (4) MRONJ pathophysiology has to be the sole aim of the study and (5) associations to MRONJ must be made in the discussion.

Types of studies to be excluded

Studies to be excluded: (1) osteonecrosis of the jaw not related to medication, (2) osteonecrosis in other body parts, (3) Studies including bisphosphonates other than zoledronate/zoledronic acid, (4) zoledronate/zoledronic acid in combination with other medications, (5) systematic reviews, etiological studies, case series and reports, consensus reports, letters, editorials, doctoral theses, pilot studies, and only abstracts, (6) studies investigating possible risk factors (i.e different diseases), (7) comparative studies between different kinds of bisphosphonates.

Condition or domain under study

Medication-related osteonecrosis of the jaw.

Population

Human cells

Interventions

Zoledronate/Zoledronic acid's effects on cellular mechanisms in bone remodelling and wound healing.

Control

None

Outcome

Medication-related osteonecrosis of the jaw diagnosed according to American Association of Oral and Maxillofacial Surgery (AAOMS) criteria (previously known as bisphosphonate-related osteonecrosis of the jaw)

Context

Years considered: The first mention of MRONJ in literature dates back to 2003; therefore, there is a small chance of identifying publications discussing the pathophysiology before that.
Language: publications in the English language only.

Primary outcome(s)

The primary outcome of this review is to evaluate and summarise what is currently known about the pathogenesis of MRONJ induced by the active substance zoledronic acid to provide a better understanding of MRONJ for practicing clinicians.

Data extraction (selection and coding)

Study selection
Titles and abstracts of identified publications from the conducted searches will be reviewed for eligibility, based on inclusion and exclusion criteria mentioned, by two independent investigators, A.A. and A.D. Publications that do not meet the eligibility criteria will be excluded from the study. Inconsistencies will be resolved by discussion and consensus between the two investigators.

Data extraction

The two investigators—A.A and A.D—independently collected data from the selected publications. The following data were collected and documented in tables: authors and year of publication, cell type, methods used, relevant reported findings.

Risk of bias (quality) assessment

The objective of this systematic review is to investigate and summarise the cellular pathophysiology of MRONJ initiated by zoledronate/zoledronic acid. It has been proven difficult to find an assessment model that correlates and is validated to the type of studies (cell studies/in vitro) included in this systematic review. Therefore the risk of bias assessment was determined using a variation based on the ToxR tool.

Strategy for data synthesis

The two investigators—A.A and A.D—independently collected data from the selected publications. The data collected from the included publications were compiled and summarised. The potential cellular mechanisms of the pathophysiology of MRONJ induced by zoledronate/zoledronic acid were investigated and documented based on the present knowledge and published research.

Funding sources/sponsors

None

Conflicts of interest

None

Table S2. Excluded articles.

	Author	Title	Year	Reason for exclusion
1	Scheller et al.	Bisphosphonates inhibit expression of p63 by oral keratinocytes	2011	Cells taken from patients treated with non-specified bisphosphonates
2	Hadaya et al.	Development of Medication-Related Osteonecrosis of the Jaw After Extraction of Teeth with Experimental Periapical Disease	2019	No pathological cellular mechanisms involved, only histological features.
3	Polidoro et al	Effects of bisphosphonate treatment on DNA methylation in osteonecrosis of the jaw	2014	Multiple bisphosphonates
4	Castro et al.	Histatin-1 counteracts the cytotoxic and antimigratory effects of zoledronic acid in endothelial and osteoblast-like cells.	2019	Includes MC3T3-E1 murine preosteoblasts
5	Agis et al.	Is zoledronic acid toxic to human periodontal fibroblasts?	2010	No association to medication-related osteonecrosis in the jaw was made in the discussion
6	Kyrgidis et al.	Increased CD14+ and decreased CD14- populations of monocytes 48 h after zolendronic acid infusion in breast cancer patients.	2017	Multiple bisphosphonates
7	Wehrhan et al.	Msx-1 is suppressed in bisphosphonate-exposed jaw bone analysis of bone turnover-related cell signalling after bisphosphonate treatment	2011	Multiple bisphosphonates
8	Mozzati et al.	Oral mucosa produces cytokines and factors influencing osteoclast activity and endothelial cell proliferation, in patients with osteonecrosis of jaw after treatment with zoledronic acid	2013	Not specified if patients were treated with other bisphosphonates
9	Movila et al.	Possible pathogenic engagement of soluble Semaphorin 4D produced by $\gamma\delta$T in medication-related osteonecrosis of the jaw (MRONJ).	2016	Multiple bisphosphonates
10	Basso et al.	Response of a co-culture model of epithelial cells and gingival fibroblasts to zoledronic acid	2016	No association to medication-related osteonecrosis in the jaw was made in the discussion
11	Elsayed et al.	Removal of matrix-bound zoledronic acid prevents post-extraction osteonecrosis of the jaw by rescuing osteoclast function.	2018	Prophylactic measures
12	Gao et al.	Zoledronic acid suppressed angiogenesis and osteogenesis by inhibiting osteoclasts formation and secretion of PDGF-BB.	2017	Murine cells
13	Kim et al.	Zoledronic acid Enhances Osteocyte-Mediated Osteoclast Differentiation by IL-6/RANKL Axis.	2019	Murine cells
14	Muratsu et al.	Zoledronic acid enhances lipopolysaccharide-stimulated proinflammatory reactions through controlled expression of SOCS1 in macrophages.	2013	Murine cells

15	Kaneko et al.	Zoledronic acid exacerbates inflammation through M1 macrophage polarization.	2018	No association to medication-related osteonecrosis in the jaw was made in the discussion

Table S3. Methods used in the included publications.

Article	Methods used
A1 Scheper et al.	- Direct microscopical observation (physical signs of apoptosis)<br>- Rhodamine assay (physical signs of apoptosis)<br>- TUNEL staining (confirmation of apoptosis)<br>- Vital stain (confirmation of apoptosis)<br>- Flow cytometry and annexin V studies (confirmation of apoptosis)<br>- MTS cell proliferation assay (cell proliferation)<br>- Statistical analysis (t-test or Student's t-test, $p < 0.05$, means ± SEM)
A2 Ravosa et al.	- Cell apoptosis assay (annexin V–FITC and propidium iodide)<br>- MTS cell proliferation assay (cell viability/proliferation)<br>- Wound scratch assay (migration)<br>- Real time PCR (type-1 collagen expression)<br>- Immunofluorescent staining (enzymatic activity, loss of collagen deposition)<br>- Gelatin zymography (MMP activity)<br>- Statistical analysis (ANOVA, T-test, mean ± SEM, $p<0.05$)
A3 Pourgonabadi et al.	- Flow cytometry (apoptosis)<br>- MTT Assay (cell proliferation)<br>- Immunofluorescent staining (morphological observation)<br>- Western blotting analysis (expression levels of pro-apoptotic and anti-apoptotic proteins)<br>- Statistical analysis (post hoc Dunnett multiple comparison tests, $p < 0.05$, means ± SEM)
A4 Scheper et al.	- Direct microscopic observation (physical signs of apoptosis)<br>- TUNEL staining (confirmation of apoptosis)<br>- Flow Cytometry and Annexin V Studies (apoptosis)<br>- Immunofluorescence analysis<br>- Cell proliferation assay (MTS or coulter counter)<br>- RT2 Profiler tm PCR Array (gene expression)<br>- Western blotting analysis (apoptosis gene array)<br>- Statistical analysis (t-test or Student's t-test, $p < 0.05$)
A5 Thibaut et al.	- Microscopy analyses (cell morphology)<br>- MTT assay (cell proliferation)<br>- Acid phosphatase assay (cell proliferation, 3D)<br>- Western blotting<br>- Statistical analysis (ANOVA, Fisher's test, $p < 0.05$, means standard deviation, SD)
A6 Wang et al.	- Flow cytometry and annexin V studies (apoptosis)<br>- Cell counting kit-8 (CCK-8) assay (cell viability)<br>- Wound scratch assay (cell migration)<br>- Western blotting

	- Angiogenesis assay<br>- Cell migration assay<br>- Statistical analysis (ANOVA; Fisher's Test, $p < 0.05$)
A7 Saracino et al.	- Cell viability with LDH (cell proliferation and viability)<br>- Real-time<br>- Flow cytometry (apoptosis)<br>- ELISA analysis<br>- Statistical analysis (Variance analysis and Newman–Keuls test, $p < 0.05$, means ±SD)
A8 Lang et al.	- Microscopical observation (cell morphology)<br>- Flow cytometry and annexin V studies (apoptosis)<br>- MTT assay (cell viability)<br>- Wound scratch assay (cell migration)<br>- Western blotting analysis (cell proliferation, migration, and apoptosis)<br>- Flow cytometry (progression of cell cycle)<br>- Statistical analysis (t-test, Mean (SEM), $p < 0.05$)
A9 Anitua et al.	- ELISA cell death detection kit (apoptosis)<br>- CYQUANT cell proliferation assay (cell proliferation and viability)<br>- Western blotting (NF-κBs expression levels)<br>- Statistical analysis (ANOVA and the Tamhane, Bonferroni-corrected post hoc tests or t test, $p < 0.05$)
A10 Lu et al.	- Microscopical observation (apoptotic morphology)<br>- Flow cytometry and annexin V studies (cell apoptosis)<br>- MTT assay (cell viability)<br>- RT-qPCR<br>- Western blotting analysis<br>- Green fluorescent protein (GFP)-LC3 adenovirus assay<br>- Statistical analysis (One-way analysis of variance, Tukey's multiple comparison post-hoc test, $p < 0.05$, mean ± standard error)
A11 Komatsu et al.	- Cell viability assay (cell viability)<br>- RT-qPCR (TGF-**β**1 and TGF-**β**2 expression levels)<br>- Immunofluorescence analysis (Type 1 collagen levels)<br>- Flow cytometric analysis (TGF-**β**1 and TGF-**β**2 expression levels)<br>- Western blotting<br>- Cell migration assay<br>- Statistical analysis (Student's t-test, $p < 0.01$, mean ± SD)

Table S4. Detailed presentation of results regarding epithelial cells. (* = significant).

	ZA-concentration (µM)		Apoptosis	Cell death	Proliferation	Viability	Migration
A1	0.5	Non-chelated	-	-	ns	-	-
		Chelated	9.88% increase (24h)*	-	-	-	-
	1	Non-chelated	1.75% increase (24h)	-	ns	-	-
		Chelated	9.69% increase (24h)*	-	12% decrease (24h)*	-	-
	3	Non-chelated	5.27% increase (24h)	few initiating cell death (24h)	ns	-	-
		Chelated	11.22% increase (24h)*	ring of dead cells (24h)	14% decrease (24h)*	-	-
	5	Non-chelated	5.79% increase (24h)	few initiating cell death (24h)	ns	-	-
		Chelated	12.91% increase (24h)*	ring of dead cells (24h)	19.4% decrease (24h)*	-	-
	10	Non-chelated	8.32% increase (24h)	-	ns	-	-
		Chelated	12.83% increase (24h)	-	19.9% decrease (24h)*	-	-
A2	5		-	-	ns (48h)	ns (48h)	-
	10		ns (48h)	-	decreased (48h)*	decreased (48h)*	ns (5h)<br>enhanced migration (10-30h)*
	30		minimal <10% (48h)*	-	decreased (48h)*	decreased (48h)*	-
	50		minimal <10% (48h)*	-	decreased (48h)*	decreased (48h)*	-

	75		ns (48h)	-	decreased (48h)*	decreased (48h)*	-
	100		minimal <10% (48h)*	-	decreased (48h)*	decreased (48h)*	-
	300		-	-	decreased (48h)*	decreased (48h)*	-
	0.25		17.84 fold increase (24h)*	ns	ns	-	-
	0.5		18.98 fold increase (24h)*	ns<br>cell damage (14-24h)	ns	-	-
A4	1		22.23 fold increase (24h)*	ns<br>cell damage (10-24h)	45.2% & 24% decrease (24h)*	-	-
	3		35.62 fold increase (24h)*	initial cell damage (6h)<br>cell damage (10-24h)	83.9% & 72% decrease (24h)*	-	-
	5		ns	-	-	50% inhibition (72h)*	decreased (72h)*
A6	50		<10% (72h)*	-	-	50% inhibition (72h)*	-
	100		<10% (72h)*	-	-	50% inhibition (72h)*	-
A7	5		35% increase (48h)*	-	12.1% inhibition (48h)*	-	-
	50		182% increase (48h)*	-	37.8% inhibition (48h)*	-	-

Table S5. Detailed presentation of results regarding fibroblasts. (* = significant).

	ZA-concentration (μM)		Apoptosis	Cell death	Proliferation	Viability	Migration
A1	0.5	Non-chelated	-	-	-	-	-
		Chelated	3.89% increase (24h)	-	-	-	-
	1	Non-chelated	2.97% increase (24h)	-	-	-	-
		Chelated	3.88% increase (24h)	-	-	-	-
	3	Non-chelated	3.19% increase (24h)	-	-	-	-
		Chelated	3.98% increase (24h)	ring of dead cells (24h)	-	-	-
	5	Non-chelated	3.42% increase (24h)	few cells initiating cell death (24h)	-	-	-
		Chelated	4.97% increase (24h)	ring of dead cells (24h)	-	-	-
	10	Non-chelated	3.39% increase (24h)	-	-	-	-
		Chelated	5.3% increase (24h)	-	-	-	-
A2	5		-	-	ns	ns	-
	10		-	-	decreased (24h)*	decreased (24h)*	ns (10-30h)<br>delayed (40-70h)*
	30		-	-	decreased (24h)*	decreased (24h)*	-
	50		-	-	decreased (24h)*	decreased (24h)*	-
	75		-	-	decreased (24h)*	decreased (24h)*	-
	100		-	-	decreased (24h)*	decreased (24h)*	-

	300		-	-	decreased (24h)*	decreased (24h)*	-
A3	0.2		-	-	ns	-	-
	0.4		-	-	ns	-	-
	0.8		-	-	ns<br>decreased (7d)*	-	-
	1.5		-	-	decreased to 84±1% (72h-7d)*	-	-
	3		-	-	decreased to 80±2% (72h)*	-	-
	6		-	-	decreased to 77±1% (72h)*	-	-
	12		-	-	decreased to 71±2.5% (72h)*	-	-
	25		-	-	decreased to 62±1.2% (72h)*	-	-
	50		-	-	decreased to 58±2% (72h)*	-	-
	100		-	-	decreased to 42±1% (72h)*	-	-
A4	0.25		ns (tunel staining), 1.99 fold increase (24h)*	ns	ns	-	-
	0.5		apoptosis shown, 3.11 fold increase (24h)*	cell damage (14h)*	ns	-	-

	1		apoptosis shown, 5.27 fold increase (24h) *	cell damage (10h)*	45.7% & 23.6% decrease (24h)*	-	-
	3		apoptosis shown, 6.25 fold increase (24h)*	initial damage (6h)*	51.1% & 31.3% decrease (24h)*	-	-
			-	cell damage (10h)*	-	-	-
A9	0.1		-	-	-	ns	-
	1		-	-	-	ns	-
	2		-	-	-	ns	-
	3		-	-	-	ns	-
	4		-	-	-	decrease in DNA quantification 83±14 ng/mL (48h), 74±2 ng/mL (96h)*	-
	5		-	-	-	decrease in DNA quantification 68±14 ng/mL (48h), 66±8 ng/mL (96h)*	-
	7.5		ns	-	-		-
	10		increased (48h)*	-	-	decrease in DNA quantification 48±7ng/mL (48h), 26±8 ng/mL (96h)*	-
	12.5		increased (48h)*	-	-	-	-

	15		increased (48h)*	-	-	-	-
	20		increased (48h)*	-	-	-	-
A11	0.0147		-	-	-	ns	-
	0.147		-	-	-	ns	-
	1.47		-	-	-	ns	-
	14.7		-	-	-	35% suppression (48h)*	-
	147		-	-	-	58% suppression (48h)*	-

Table S6. Detailed presentation of results regarding osteoblasts. (* = significant).

	ZA-concentration (µM)	Apoptosis	Proliferation	Viability
A5	0.1	-	ns (3, 10d)	ns (3d)
				increased to 114.5±9.0% (10d)*
	10	-	decreased to 78.4±7.4% (3d)*	ns (3d)
			decreased to 40.4±9.7% (10d)*	decreased to 80.4±12.3% (10d)*
A7	5 CM	-	decreased by 20.8% (48h)*	ns (48h)
	5	-	decreased (48h)*	ns (48h)
	50 CM		decreased by 60% (48h)*	ns (48h)
A9	1	ns (48h)	-	-
	5	ns (48h)	-	-
	10	ns (48h)	-	-
	15	ns (48h)	-	-
	20	increased (48h)*	-	-
	25	increased (48h)*	-	-

	50	increased (48h)*	-	-
	100	increased (48h)*	-	-

Table S7. Detailed presentation of results regarding endothelial cells. (* = significant).

	ZA-concentration (μM)	Apoptosis	Viability	Migration
A6	5	ns (48h)	ns (24h)<br>decreased (48h)*	inhibited (48h)*
	50	increased (48h)*	ns (24h)<br>decreased (48h)*	-
	100	increased (48h)*	ns (24h)<br>decreased (48h)*	-
A8	0.23	-	ns (24h)	-
	0.69	-	ns (24h)	-
	2.06	-	decreased (24h)*	-
	6.17	-	decreased (24h)*	-
	15	ns (24h)<br>increased (48h)*	-	decreased (24h)*
	18.25	-	decreased (24h)*	-
	50	ns (24h)<br>increased (48h)*	-	decreased (24h)*<br>-
	55.56	-	decreased (24h)*	-
	150	ns (24h)<br>increased (48h)*	-	decreased (24h)*
	166.67	-	decreased (24h)*	-
	500	-	decreased (24h)*	-
A10	25	increased (48h)*	ns (48h)	-
	50	increased (48h)*	decreased (48h)*	-
	75	increased (48h)*	decreased (48h)*	-

	100	increased (48h)*	decreased to 68.4±5.8% (48h)*	-

Table S8. Detailed presentation of results regarding dental pulp stem cells. (* = significant).

	ZA-concentration (μM)	Apoptosis	Proliferation	Viability
A3	0.2	-	ns (24/48/72h/7d)	-
	0.4	-	ns (24/48/72h/7d)	-
	0.8	increased to 27±8% (72h)*	ns (24/48h)	-
			decreased to 68±3% (72h)*	
			decreased (7d)*	
	1.5	increased to 43±8% (72h)*	ns (24, 48h)	decreased (72h)*
			decreased to 67±5% (72h)*	
			decreased (7d)*	
	3	increased to 46±6.5% (72h)*	ns (24, 48h)	-
			decreased to 67±6% (72h)*	
			decreased (7d)*	
	6	increased to 60±2% (72h)*	ns (24/48h)	-
			decreased to 68±4.5% (72h)*	
			decreased (7d)*	
	12	increased to 70±6.5% (72h)*	ns (24/48h)	-
			decreased to 67±5% (72h)*	
			decreased (7d)*	
	25	increased to 84±2% (72h)*	ns (24/48h)	decreased (72h)*
			decreased to 61±4% (72h)*	
			decreased (7d)*	
	50		ns (24, 48h)	-

		increased to 88±1% (72h) *	decreased to 51±5% (72h)*	
			decreased (7d)*	
	100	-	ns (24h)	decreased (72h)*
			decreased (48h)*	
			decreased to 33±4% (72h)*	
			decreased to 8±2% (7d)*	
